# Biofertilizer Potential of Aquatic Macrophytes in Improving Soil Quality and *Urochloa decumbens* Growth: An Integrated Approach Using rSQS and rPPS

**DOI:** 10.3390/plants15121788

**Published:** 2026-06-10

**Authors:** Ramon Hernany Martins Gomes, Carlos Roberto de Toffoli, Pedro Luis da Costa Aguiar Alves, Robinson Luiz Pitelli, Rinaldo José da Silva Rocha, Felipe Pinheiro da Cruz, Antônio Manoel Matta dos Santos Lameirão, Arilson José de Oliveira Júnior, Esther Camilo dos Reis, Rafael Plana Simões, Robinson Antonio Pitelli

**Affiliations:** 1 School of Agriculture, São Paulo State University (UNESP), 3780 Universitaria Avenue, Botucatu 18610-034, SP, Brazil; r.gomes@unesp.br (R.H.M.G.); arilson.oliveira@unesp.br (A.J.d.O.J.); e.reis.esib@esib.butantan.gov.br (E.C.d.R.); 2 School of Agrarian and Veterinary Sciences, São Paulo State University (UNESP), Via de Acesso Prof. Paulo Donato Castellane s/n, Jaboticabal 14884-900, SP, Brazil; cr.toffoli@unesp.br (C.R.d.T.); pl.alves@unesp.br (P.L.d.C.A.A.); 3 Ecosafe Agricultura e Meio Ambiente SS Ltda., 856 Rua Monteiro Lobato St., Jaboticabal 14870-410, SP, Brazil; rlpitelli@ecosafe.agr.br; 4 LIGHT Energia S.A. (Sociedade Anônima), 168 Marechal Floriano Avenue, Rio de Janeiro 20080-002, RJ, Brazil; rinaldo.rocha@light.com.br (R.J.d.S.R.); felipe.cruz@light.com.br (F.P.d.C.); antonio.lameirao@light.com.br (A.M.M.d.S.L.)

**Keywords:** aquatic macrophytes, biofertilizer, soil quality index, plant quality index, soil fertility, *Urochloa decumbens*

## Abstract

The excessive proliferation of aquatic macrophytes in Brazilian reservoirs generates large amounts of biomass that must be removed to ensure water use and energy generation. This material is usually treated as waste, but its recycling as a biofertilizer could mitigate disposal problems while improving soil and crop productivity. To test this potential, we conducted a 4 × 3 factorial experiment using four macrophyte species (*Salvinia auriculata* Aubl., *Myriophyllum aquaticum* (Vell.) Verdc., *Egeria densa* Planch. and *Pistia stratiotes* L.) collected from the Santana Reservoir, applied at three incorporation doses (5, 10 and 20 t ha^−1^ dry matter) plus a control in pots with *Urochloa decumbens* seedlings. Soil chemical properties and plant growth attributes were integrated into the relative Soil Quality Score (rSQS) and relative Plant Performance Score (rPPS). Both scores increased with macrophyte incorporation, though responses depended on species and dose. A significant positive relationship was found between rSQS and rPPS (R^2^ = 0.73; ρ = 0.85), indicating that improvements in integrated soil quality were generally associated with better plant performance. While most treatments improved score values, ELDDE at 20 t ha^−1^ showed a further increase in rSQS but no proportional gain in rPPS, which was numerically lower than at 10 t ha^−1^ but not statistically different. This pattern indicates that increases in integrated soil quality may not always translate linearly into plant performance. These results support the potential of selected macrophyte materials as organic amendments and highlight the need for biomass characterization, contaminant monitoring and field validation before practical recommendation.

## 1. Introduction

In pristine environments, macrophytes provide crucial environmental services within continental waterbodies. However, in environments under significant anthropogenic influence, some macrophyte species, particularly exotic ones, develop large populations. These outcompete native species and alter the dynamics of the biocoenosis, impacting water use and the waterbody itself, leading to economic, environmental and social detriments [1]. In numerous waterbodies, extensive macrophyte colonization necessitates substantial control interventions to maintain their social, economic and/or environmental functions [2]. In Brazil, herbicide application is not an option for macrophyte control. The predominant method is mechanical harvesting, where plants are removed from the water column and deposited on land. While this method offers rapid area clearing, its resilience is low due to the high growth rate of macrophytes. Furthermore, it generates a large quantity of biomass that requires environmentally safe disposal [3].

According to Brazilian legislation, aquatic macrophyte biomass managed as solid waste/reject must be disposed of in landfills and requires environmental licensing for urban solid waste disposal systems (https://www.planalto.gov.br/ccivil_03/_ato2007-2010/2010/lei/l12305.htm, accessed on 9 June 2026). However, plant material harvested from natural aquatic environments should not be automatically classified as solid waste, it is only considered as such under specific conditions, such as in tertiary wastewater treatment systems. Within the framework of the circular bioeconomy, harvested macrophyte biomass should not be treated solely as waste but as a potential resource that can be reintegrated into agricultural systems, supporting nutrient recycling and closing nutrient loops between aquatic and terrestrial ecosystems [4,5]. Several studies have demonstrated that using this material as a biofertilizer, through direct application, composting, vermicomposting or liquid formulations, is a viable alternative [5,6,7,8], generally without heavy metal levels that pose a risk of soil contamination [1,9,10,11]. Consequently, this represents an environmentally sustainable and intelligent strategy for utilizing this material [9,12].

The potential for nutrient recycling from macrophyte biomass is desirable, but the safety and agronomic value of the resulting amendments depend strongly on the source material. Macrophytes harvested from natural waterbodies, such as reservoirs, typically reflect geogenic sediment chemistry and may present low contaminant risk [9,11,13]. In contrast, biomass collected from tertiary urban or industrial effluent treatment systems may contain elevated concentrations of heavy metals or other potentially toxic elements derived from the wastewater [14]. In such cases, the recycling process may inadvertently transfer undesirable contaminants to agricultural soils. Therefore, a rigorous chemical characterization of the biomass is essential before its use as a soil amendment and source-dependent risk assessment should guide decisions on whether the material is suitable for agricultural reuse or requires disposal as urban solid waste [11,14,15].

Soil quality directly correlates with agricultural productivity potential. Therefore, developing and applying objective criteria for its assessment is essential. However, the inherent complexity and specificity of each soil make it challenging to translate its characteristics into measurable attributes that accurately reflect its functional state. Depending on the specific function to be evaluated, a broad range of indicators may be considered. This requires a meticulous selection of attributes that are simultaneously relevant to the evaluated function, measurable and sensitive to spatial or temporal variations [16,17]. When evaluating complex organic amendments, such as macrophyte biomass, that may simultaneously affect nutrient availability, soil chemical balance, microbial activity and plant growth [18,19], integrated assessment frameworks are particularly valuable. Composite indicators that combine multiple soil attributes (e.g., pH, nutrient concentrations, organic matter, possible contaminants) into a single numerical scale enable systematic comparison of amendment effects and facilitate the identification of management practices that enhance or compromise soil sustainability [20,21]. Similarly, integrating plant attributes can allow the biological response to these amendments to be evaluated in parallel with changes in soil quality.

These approaches typically merge physical, chemical and biological indicators through normalization and weighting functions, allowing the identification of management practices that enhance or compromise soil sustainability and plant performance [22]. Building on these principles, we implemented relative scoring systems to assess both soil quality and plant growth, referred to here as the relative Soil Quality Score (rSQS) and the relative Plant Performance Score (rPPS).

This study aimed to determine the potential benefits and risks of using aquatic macrophytes as biofertilizers. For this purpose, we evaluated biomass from predominant species found in the Santana Reservoir (Piraí, RJ, Brazil), analyzing their chemical composition (including potentially toxic elements) and the resulting effects on soil properties and the growth of the forage plant *Urochloa decumbens*. The selected species included two submerged macrophytes and two free-floating macrophytes. Submerged and floating macrophytes may differ in structural composition (cellulose, hemicellulose and lignin content), nutrient stoichiometry and heavy metal uptake patterns, which can influence decomposition dynamics, nutrient release rates and soil–plant responses [13,23,24]. Therefore, we expected that macrophyte incorporation could improve soil quality and plant performance, but that the balance between agronomic benefits and potential risks would depend on species identity and application dose. To comprehensively assess the agronomic and environmental implications of this practice, we applied adapted methodologies for calculating the rSQS and rPPS, enabling a comparative interpretation of soil and plant responses under different treatments.

## 2. Materials and Methods

**Figure 1:**
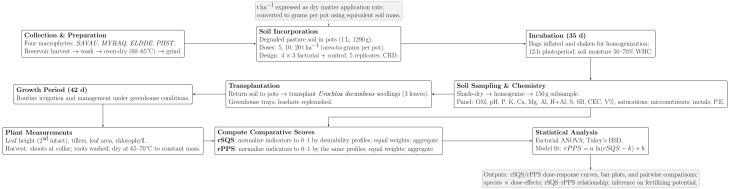
Schematic representation of the experimental workflow, from macrophyte collection and sample preparation to soil incubation, transplantation of *Urochloa decumbens*, growth evaluation, and calculation of rSQS and rPPS.

This study was conducted in sequential stages designed to evaluate the potential of aquatic macrophytes as organic fertilizers. Initially, biomass from four macrophyte species was collected, dried and ground. The material was then incorporated into degraded pasture soil at three doses (5, 10, and 20 t ha^−1^), followed by a 35-day incubation period. After incubation, soil samples were analyzed for chemical properties, and *Urochloa decumbens* seedlings were transplanted to the treated soils. Plant growth was monitored for 34 days, after which morphological and dry mass accumulation were measured. Based on these data, rSQS and rPPS were calculated and statistical analyses were applied to evaluate treatment effects. The experimental flow is summarized in Figure 1.

### 2.1. Study Site, Collection, and Sampling of Macrophyte Plants

The aquatic macrophytes used in this study were *Salvinia auriculata* Aubl. (SAVAU), *Myriophyllum aquaticum* (Vell.) Verdc. (MYRAQ), *Egeria densa* Planch. (ELDDE), and *Pistia stratiotes* L. (PIIST). These species were selected because they are ecologically relevant in the Santana Reservoir and represent contrasting biological forms, including two submerged species, ELDDE and MYRAQ, and two free-floating species, PIIST and SAVAU [13]. Additional information on the comparative features of these species is provided in Appendix A.1.

The macrophytes were collected in the Santana Reservoir in June 2023. This reservoir is located in Piraí city, approximately 80 km south of Rio de Janeiro city (Piraí, RJ, Brazil: 43°49′08″ W, 22°31′56″ S). It hosts an extensive and highly diverse community of aquatic plants, predominantly emergent species [1]. The reservoir is also characterized by being fed by water pumped from the Paraíba do Sul and Piraí rivers, between the Santa Cecília and Vigário pumping stations. This system transfers water to the Vigário Reservoir, situated 50 m above the Paraíba do Sul River, to harness a 314 m drop along the slopes of the Serra do Mar for electricity generation at the Nilo Peçanha, Fontes Nova, and Pereira Passos hydroelectric plants.

Motorized boats were used to inspect the entire length of the reservoir. Macrophytes were collected at three distinct points and placed into plastic bags. The sampling procedure involved collecting different parts of the plants, including leaves, flowers, stems and, when possible, roots. This simulated the mechanical harvesting process that would yield biomass for biofertilizer production. The plants were washed with water from the reservoir itself, as the study aimed to evaluate the potential of these species as organic fertilizer within a mechanized harvesting system. In the laboratory, samples were oven-dried with forced air circulation, maintained between 60 and 65 °C, until a constant weight was achieved. Finally, the dried material was ground in a “Willey” type micromill. Although the biomass was dried and ground in the laboratory to ensure experimental control and standardization of doses on a dry matter basis, it is important to note that, in practice, aquatic macrophytes are harvested and applied fresh, typically containing around 90–95% water.

### 2.2. Biofertilization Experiments Using Macrophyte Plants

An experiment was conducted to evaluate the effects of incorporating three doses of dried macrophyte material from four aquatic species on the chemical characteristics of soil collected from a degraded pasture area (0–10 cm surface layer; sandy loam texture; apparent density of 1.29 g cm^−3^). A completely randomized experimental design was adopted with five replicates. Treatments were arranged in a 4 × 3 factorial scheme, comprising four macrophyte species (SAVAU, MYRAQ, ELDDE, and PIIST) and three incorporation doses (5, 10, and 20 t ha^−1^ dry matter). In addition to the factorial treatments, a control treatment without macrophyte incorporation was included. The unit t ha^−1^ was adopted because it is the standard agronomic expression of application rate and allows direct comparison with field conditions. In the pot experiment, the field rates were converted to pot-scale amounts based on an equivalent soil mass calculation, considering a 0–10 cm soil layer for 1 ha. The detailed conversion procedure is described in Appendix A.1.

Experimental units consisted of pots containing 1 L of air-dried fine soil, corresponding to 1290 g, before the incorporation of the dried macrophyte material. Detailed chemical analyses of the soil under each condition are provided as Supplementary Material, which also includes a temporal series of the chemical composition of the macrophytes. The control treatment serves as a baseline from which the soil’s initial chemical composition can be inferred. The amounts of dried material corresponding to each dose were incorporated into the soil in 10 L plastic bags. These bags were previously inflated and vigorously shaken to ensure homogenization. Incubation was carried out in a climate-controlled room with a 12 h photoperiod. Soil moisture was maintained between 50% and 70% of its water-holding capacity, according to the applied dose. The incubation period was set to 35 days, following the results reported in [25,26].

After incubation, the content of each pot was dried in the shade, homogenized and sampled (150 g) for chemical analyses. The remaining soil was returned to its respective pots, where *Urochloa decumbens* seedlings with three or four fully developed leaves, previously grown in vegetable trays, were transplanted. Additional details on the seed source and seedling production are provided in Appendix A.1. The pots were maintained in a greenhouse, arranged on individual trays, allowing for the replenishment of leached water with each subsequent irrigation. The plant growth period was 34 days.

Before harvest, the height of the second largest intact leaf was recorded and the number of tillers per plant was counted. Chlorophyll content was measured using a portable chlorophyll meter (Clorofilog CFL1030, Falker Automação Agrícola Ltda., Porto Alegre, Brazil). Leaf area was determined using a benchtop leaf area meter equipped with 128 LEDs (LI-3100C, LI-COR Biosciences, Lincoln, NE, USA). Aerial parts were cut at collar level and separated into leaves and stems. Roots were separated from the soil using controlled water jets. Leaves, stems, and roots were oven-dried separately with forced air circulation at 65–70 °C until reaching constant weight. Weighing was performed on an analytical balance with centigram precision.

### 2.3. Relative Soil Quality Score (rSQS)

The rSQS was calculated to integrate soil chemical and physicochemical attributes into a single comparative measure. The score ranges from 0 to 1, where values closer to 0 indicate poorer relative soil quality within the evaluated dataset, whereas values closer to 1 indicate better relative soil quality. Therefore, the rSQS should be interpreted as a relative index for comparing treatments rather than as an absolute measure of soil quality.

The variables used for rSQS calculation included organic matter (OM), pH, phosphorus (P), potassium (K^+^), calcium (Ca^2+^), magnesium (Mg^2+^), aluminum (Al^3+^), potential acidity (H^+^ + Al^3+^), sulfur (S), sum of bases (SB), cation exchange capacity (CEC), base saturation (V%), aluminum saturation (Sat Al^3+^), potassium saturation (Sat K^+^), calcium saturation (Sat Ca^2+^), magnesium saturation (Sat Mg^2+^), boron (B), copper (Cu^2+^), manganese (Mn^2+^), iron (Fe^2+^/Fe^3+^), zinc (Zn^2+^), chromium (Cr^3+^), nickel (Ni^2+^), cadmium (Cd^2+^), lead (Pb^2+^) and water holding capacity (P.E.).

Each indicator was assigned to one of three desirability profiles: “more is better”, “less is better” or “optimal value”. The original values were then standardized to a 0–1 scale using profile-specific scoring functions. For simplicity and neutrality, equal weights were assigned to all indicators, and the rSQS was calculated as the weighted sum of the standardized values. The method used for score construction was adapted from [20]. The detailed equations, parameter estimation procedures, desirability profiles and final parameter values used for each indicator are provided in Appendix A.2 and Supplementary Material.

### 2.4. Relative Plant Performance Score (rPPS)

The determination of rPPS followed an approach similar to that used for rSQS calculation. The objective was to synthesize the overall plant performance under different treatments into a single comparative score, enabling the analysis of relationships between changes in soil properties and plant response. For this, the following attributes related to plant growth and vitality, measured 34 days after treatment, were used: number of tillers, leaf area, chlorophyll content, plant height, leaf dry mass, stem dry mass and root dry mass.

Each of these indicators was normalized according to its desirability profile, assuming values between 0 and 1. Subsequently, the same weighting was assigned to all attributes and the normalized values were integrated to obtain the rPPS. Additional methodological details are provided in Appendix A.2 and Supplementary Material.

### 2.5. Statistical Analysis

Treatment responses of rSQS and rPPS were graphically summarized using species-specific dose–response curves and bar plots. Dose–response curves were fitted using a logarithmic function to describe the trend of each score across application doses. For each score, a one-way ANOVA was first performed considering all treatment conditions, followed by Tukey’s HSD test for pairwise comparisons. A factorial ANOVA was then performed using only the macrophyte-amended treatments to evaluate the effects of species, dose and their interaction. The control treatment was not included in the factorial ANOVA because it was not part of the species × dose factorial design. Using the treatment means, the relationship between rSQS and rPPS was fitted using the logarithmic model described in Equation (1):

(1) $$rPPS=a\ln\left(rSQS-k\right)+b$$

where a, b and k are fitted parameters. Model fit was evaluated using R^2^ and Pearson’s correlation coefficient was calculated for the linearized relationship between rPPS and $\ln\left(rSQS-k\right)$. All statistical analyses and graphical representations were implemented using Python 3.12.3.

## 3. Results

**Figure 2:**
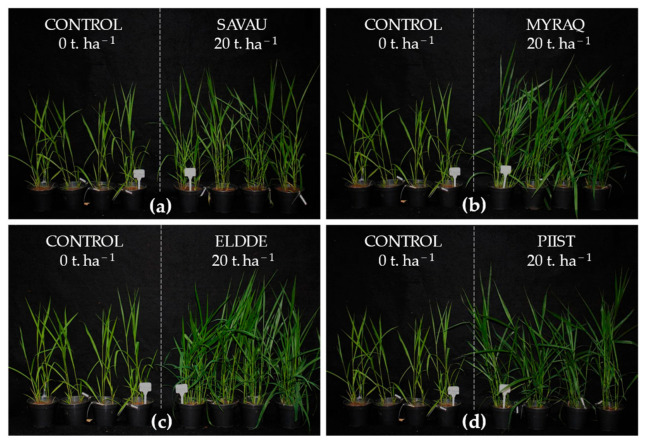
Comparative panel of *Urochloa decumbens* grown in soil with the incorporation of 20 t ha^−1^ of macrophyte material from different macrophyte species (**a**) SAVAU, (**b**) MYRAQ, (**c**) ELDDE, and (**d**) PIIST, alongside the control treatment without macrophyte incorporation (0 t ha^−1^). The photographs illustrate clear differences in plant growth among treatments, with SAVAU resulting in the lowest relative vigor, while the other species promoted greater leaf area.

Figure 2 presents a comparative panel of *Urochloa decumbens* grown in soil with the incorporation of 20 t ha^−1^ of macrophyte material from different macrophyte species, alongside the unamended control. The photographs show visible differences in plant development between the control and macrophyte-amended treatments, providing a qualitative indication that macrophyte incorporation affected plant growth. This initial visual evidence was subsequently quantified using the rPPS and interpreted in relation to the rSQS.

**Figure 3:**
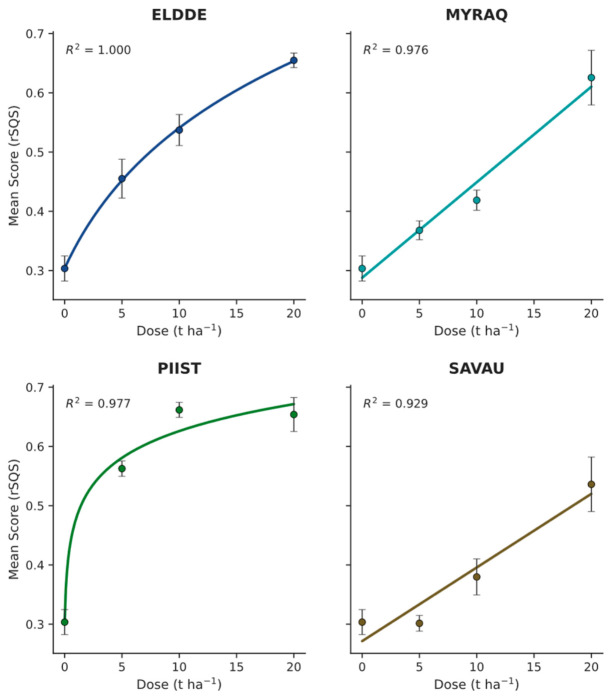
Species-specific dose–response curves of rSQS as a function of macrophyte incorporation dose. Points represent treatment means (*n* = 5), error bars indicate standard deviation and fitted curves summarize the dose–response trend within each macrophyte species.

Changes in rSQS were first analyzed across application doses within each macrophyte species. The species-specific dose–response curves showed that soil quality scores generally increased with dose, although the magnitude and shape of this response differed among species (Figure 3). ELDDE and PIIST showed a more saturating response, with larger increases at lower doses and smaller gains at higher doses, whereas MYRAQ and SAVAU exhibited a more gradual, approximately linear increase across the evaluated dose range.

A one-way ANOVA considering all treatment conditions, including the unamended control, indicated significant differences in rSQS among treatments (p<0.0001). This result confirms that macrophyte incorporation produced measurable changes in the integrated soil quality score relative to the overall set of experimental conditions. Among the macrophyte-amended treatments, factorial ANOVA showed significant effects of species, dose, and species × dose interaction on rSQS values (Table 1), indicating that the soil response depended not only on the amount of material incorporated but also on the species used.

**Figure 4:**
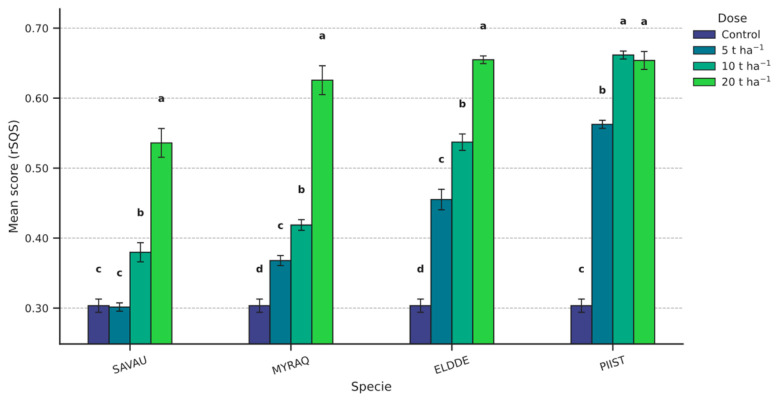
Mean rSQS values according to macrophyte species and incorporation dose. Bars represent means ± standard error (*n* = 5). Within each macrophyte species, the unamended control is shown as a reference condition, and bars sharing the same letter do not differ significantly according to Tukey’s HSD test (α=0.05).

**Figure 5:**
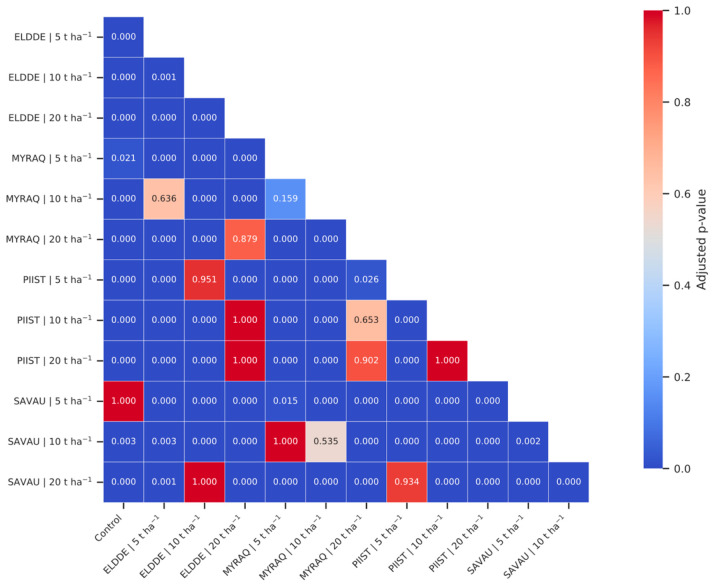
Matrix of *p*-values from Tukey’s HSD pairwise comparisons among treatment conditions for rSQS. Values indicate adjusted *p*-values associated with each pairwise comparison. For visualization purposes, *p*-values are displayed with three decimal places; therefore, values lower than 0.0005 appear as 0.000.

To complement the dose–response curves and factorial ANOVA, mean rSQS values were summarized in a bar plot with compact letter displays (Figure 4). The compact letter display summarizes Tukey’s HSD comparisons among dose levels within each macrophyte species, indicating which doses produced statistically distinct rSQS values. Only SAVAU at 5 t ha^−1^ did not differ from the unamended control, whereas all other macrophyte-amended treatments resulted in significantly higher rSQS values. The rate of increase varied clearly among species and PIIST showed an apparent saturation pattern, with no significant gain in rSQS between 10 and 20 t ha^−1^. The complete Tukey’s HSD pairwise comparison matrix is shown in Figure 5. This matrix complements the compact letter display by allowing direct visualization of specific pairwise differences among treatment conditions, including comparisons involving the control.

**Figure 6:**
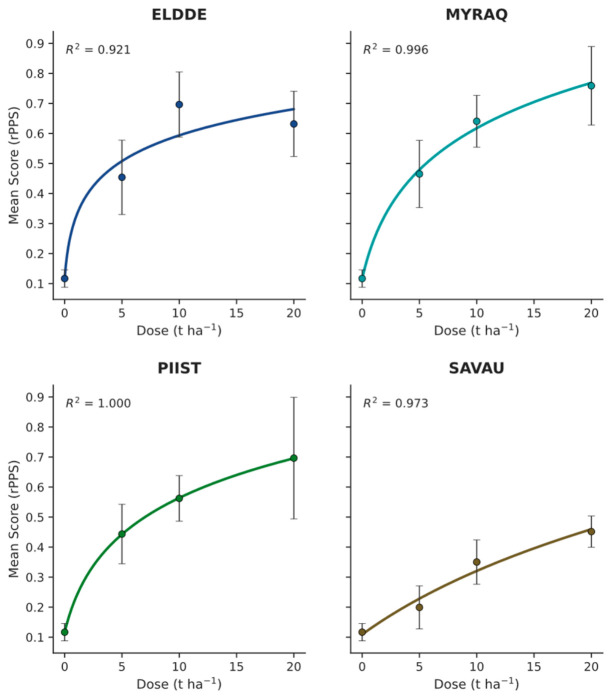
Species-specific dose–response curves of rPPS as a function of macrophyte incorporation dose. Points represent treatment means (*n* = 5), error bars indicate standard deviation and fitted curves summarize the dose–response trend within each macrophyte species.

**Figure 7:**
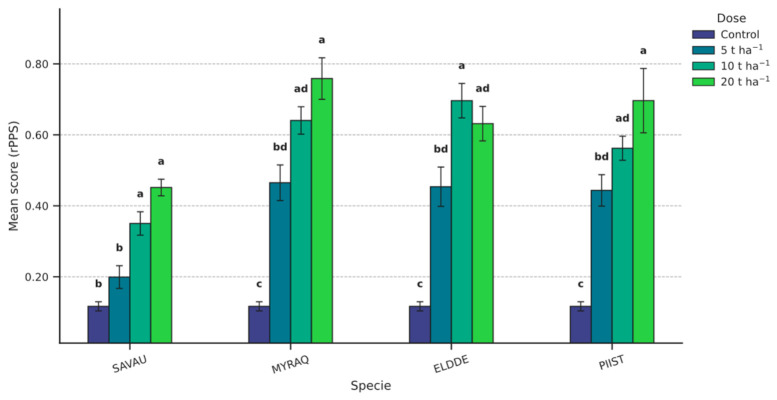
Mean rPPS values according to macrophyte species and incorporation dose. Bars represent means ± standard error (*n* = 5). Within each macrophyte species, the unamended control is shown as a reference condition, and bars sharing the same letter do not differ significantly according to Tukey’s HSD test (α=0.05).

**Figure 8:**
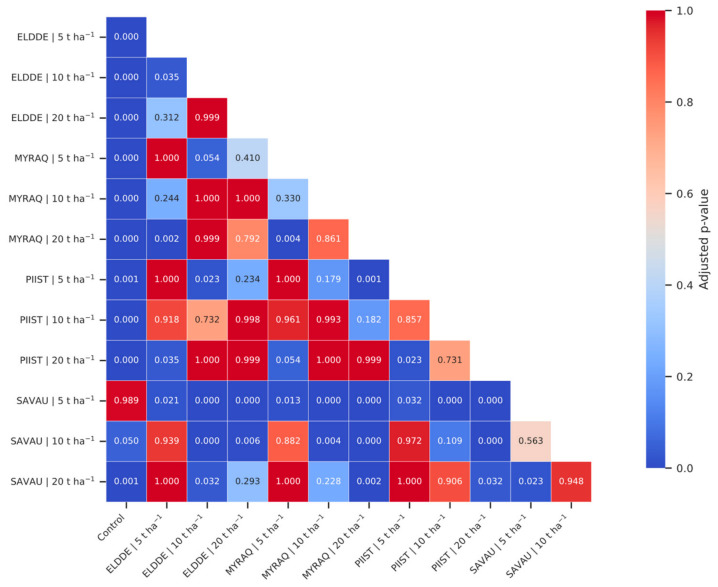
Matrix of *p*-values from Tukey’s HSD pairwise comparisons among treatment conditions for rPPS. Values indicate adjusted *p*-values associated with each pairwise comparison. For visualization purposes, *p*-values are displayed with three decimal places; therefore, values lower than 0.0005 appear as 0.000.

Similarly, the rPPS response was analyzed using species-specific dose–response curves (Figure 6). These curves showed that plant performance generally increased with macrophyte incorporation, especially between the lower dose levels and tended to approach a plateau at higher doses. SAVAU showed lower rPPS values than the other species across the evaluated dose range, indicating a weaker overall plant response to this material. A one-way ANOVA considering all treatment conditions, including the unamended control, indicated significant differences in rPPS among treatments (*p* < 0.0001). Among the macrophyte-amended treatments, factorial ANOVA showed significant effects of species and dose on rPPS values, whereas the species × dose interaction was not significant (Table 2). This indicates that plant performance depended on both the type and amount of incorporated macrophyte material, but without statistical evidence that the dose–response pattern differed among species. Mean rPPS values were summarized in a bar plot with compact letter displays (Figure 7). SAVAU at 5 t ha^−1^ was the only macrophyte-amended treatment that did not differ significantly from the unamended control. The complete pairwise comparison matrix for rPPS is presented in Figure 8, allowing the identification of specific treatment pairs with statistically significant differences in plant performance.

**Figure 9:**
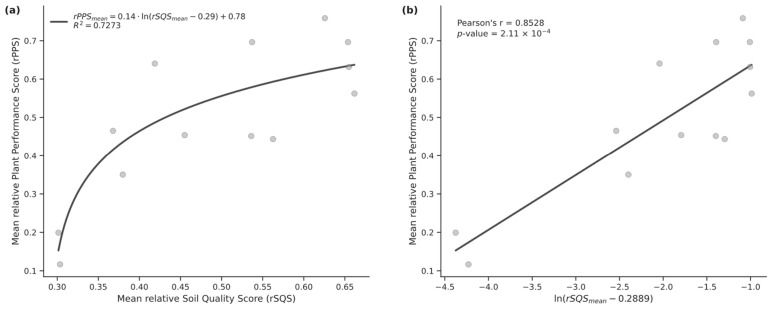
Relationship between rPPS and rSQS. (**a**) shows the direct relationship between rPPS and rSQS, with a fitted curve described by Equation (4), which had a coefficient of determination of $R^{2}=0.7273$. (**b**) shows the relationship between rPPS and the natural logarithm of the difference between rSQS and the adjusted constant *k* (ln(rSQS − 0.2889)). This relationship yielded a high Pearson correlation coefficient (ρ=0.8528;p−value=0.0002).

Figure 9 illustrates the relationship between the rPPS and rSQS. Figure 9a shows the fit of a logarithmic model given by Equation (4), which resulted in a coefficient of determination of R^2^ = 0.7273. The parameter *k*, fitted at 0.2889, approximately corresponds to the lowest rSQS values obtained in the control treatments. Figure 9b shows the relationship between rPPS and ln(rSQS−0.2889), which resulted in a high Pearson correlation coefficient (ρ). The model fit, the correlation coefficient and its statistical significance indicate a strong positive association between rSQS and rPPS, suggesting that plant performance tended to increase as integrated soil quality improved. However, the relationship was non-linear, indicating that increases in rSQS were not translated proportionally into rPPS across the full range of treatments.

The incorporation of macrophyte material altered the concentrations of all evaluated elements in the soil, with marked differences among species and application doses (Table 3). Overall, H^+^ + Al^3+^ values decreased in all amended treatments relative to the unamended control, reaching the lowest relative value under PIIST at 20 t ha^−1^, corresponding to 43.5% of the control. Among the potentially toxic elements, Mn^2+^ showed the most pronounced relative increase. The highest Mn^2+^ concentrations were observed under MYRAQ at 20 t ha^−1^ and ELDDE at 20 t ha^−1^, corresponding to 4429.0% and 3681.2% of the control, respectively. SAVAU and PIIST also increased Mn^2+^ concentrations, but to a lower extent than MYRAQ and ELDDE. Zn^2+^ also increased with dose, particularly under ELDDE and MYRAQ, reaching 420.7% and 425.9% of the control at 20 t ha^−1^, respectively.

Ni^2+^ and Cd^2+^ showed pronounced relative increases under SAVAU and PIIST, whereas Pb^2+^ reached the highest values under MYRAQ. In contrast, Cu^2+^ remained close to or below the control in most treatments, and Pb^2+^ was below the control in ELDDE at 5 t ha^−1^. These reductions may reflect changes in metal recovery during soil extraction after organic matter addition. The incorporated macrophyte material may have altered pH, ligand availability, adsorption sites and precipitation reactions, potentially shifting part of the metal pool into less extractable forms and thereby reducing the concentrations detected by the analytical procedure used.

Despite the relative increases observed for several elements, the highest absolute concentrations of Cd^2+^, Pb^2+^, Cr^3+^, Ni^2+^, Cu^2+^ and Zn^2+^ remained below the soil prevention values established by CONAMA Resolution No. 420/2009 [27]. The maximum values observed were 0.198 mg kg^−1^ for Cd^2+^, 1.254 mg kg^−1^ for Pb^2+^, 0.950 mg kg^−1^ for Cr^3+^, 1.040 mg kg^−1^ for Ni^2+^, 1.160 mg kg^−1^ for Cu^2+^ and 11.500 mg kg^−1^ for Zn^2+^, all below the corresponding prevention values of 1.3, 72, 75, 30, 60, and 300 mg kg^−1^, respectively. For Mn^2+^, no soil prevention value is provided in CONAMA Resolution No. 420/2009; therefore, the pronounced increase observed, reaching 61.12 mg kg^−1^ under MYRAQ at 20 t ha^−1^, should be interpreted as an agronomic and environmental warning rather than as a formal regulatory exceedance.

## 4. Discussion

The present study evaluated the agronomic potential and environmental implications of using aquatic macrophyte material as an organic amendment for degraded pasture soil. Overall, macrophyte incorporation improved both the rSQS and the rPPS, supporting the potential of selected macrophyte materials to contribute to soil recovery and *U. decumbens* growth under controlled experimental conditions. However, the magnitude of the response depended on macrophyte species and application dose, indicating that aquatic plant material should not be treated as a uniform amendment. This species- and dose-dependent behavior is consistent with previous evidence that macrophytes differ in nutrient composition, structural compounds, decomposition dynamics and organic matter quality, all of which may influence their agronomic performance after soil incorporation [13,23,28].

A central finding of this study was the positive relationship between rSQS and rPPS. Importantly, these indices were constructed independently: rSQS integrated soil attributes, whereas rPPS integrated plant performance traits. Therefore, the observed association indicates that improvements in the integrated soil quality score were accompanied by better plant performance, although this should not be interpreted as direct proof of a single causal mechanism. The logarithmic pattern suggests that plant performance increased more strongly at lower rSQS values and then tended toward saturation, indicating diminishing gains as soil quality improved. This behavior is agronomically plausible because, after major soil constraints are alleviated, plant performance may tend toward saturation, so additional improvements in soil quality may not result in proportional gains in plant growth. This non-linear response may also reflect factors not fully represented in rSQS, such as nutrient balance, decomposition dynamics or microbial processes.

The improvement in rSQS is consistent with the expected effects of organic amendments derived from aquatic macrophytes. These plants can accumulate nutrients in their biomass, and their incorporation into soil may contribute to nutrient recycling, organic matter input, changes in soil potential acidity and increased availability of exchangeable nutrients [1,5,12,13,15]. In the present study, all macrophyte-amended treatments reduced H^+^ + Al^3+^ relative to the unamended control, suggesting a decrease in potential acidity that likely contributed to the improvement in rSQS. In addition to direct nutrient inputs, macrophyte residues may affect microbial activity and organic matter dynamics, potentially influencing nutrient mineralization and soil biological functioning [19,23]. Nevertheless, because the present study used integrated indices, these mechanisms should be interpreted as plausible contributors rather than individually demonstrated causal pathways.

The response of ELDDE illustrates the non-proportional relationship that may occur between integrated soil quality and plant performance. At the highest dose, ELDDE further increased rSQS but did not produce a proportional increase in rPPS. Although rPPS was numerically lower at 20 t ha^−1^ than at 10 t ha^−1^, this difference was not statistically significant according to Tukey’s HSD test. In addition, the factorial ANOVA showed a non-significant species × dose interaction for rPPS, indicating that this pattern should be interpreted as descriptive rather than as evidence of a statistically distinct ELDDE dose–response pattern. Therefore, the present data do not support attributing this response to a species-specific inhibitory mechanism.

Nevertheless, this result highlights a relevant limitation of integrated soil quality scores: improvements in measured soil attributes may not capture all biological or chemical factors that influence plant performance. In the present study, the potentially toxic elements included in rSQS were already incorporated as lower-is-better indicators, and the concentrations of Cd^2+^, Pb^2+^, Cr^3+^, Ni^2+^, Cu^2+^, and Zn^2+^ remained below Brazilian soil prevention values where such limits are available. Thus, the non-proportional rSQS–rPPS pattern observed for ELDDE should be interpreted descriptively, indicating that factors beyond the measured soil attributes may deserve attention when interpreting plant performance. Among these possible factors, organic compounds released during macrophyte decomposition are particularly relevant, since plant-derived phenolic compounds can act as allelochemicals and extracts from aquatic or semi-aquatic plants may affect germination and plant growth in a concentration-dependent manner [29,30]. In addition, previous work with *Egeria densa* extracts reported dose-dependent effects on crop growth [31]. However, because such compounds were not measured in the present study, their role cannot be inferred from the current data and should be evaluated in future studies using targeted chemical profiling, decomposition assays and phytotoxicity tests.

The evaluation of potentially toxic elements requires distinguishing metal accumulation capacity from actual soil contamination risk. Aquatic macrophytes are known to accumulate metals and are widely discussed as tools for phytoremediation of contaminated aquatic environments [24,32,33]. However, the ability of a species to accumulate metals does not automatically imply that its agronomic reuse will contaminate soil. Risk depends on the source environment, plant species, contaminant concentration in the biomass, application dose, soil properties and metal availability [34]. In this study, several elements increased relative to the control, especially Mn^2+^, which reached 61.12 mg kg^−1^ under MYRAQ at 20 t ha^−1^, equivalent to 4429.0% of the control. This increase deserves attention because Mn can become phytotoxic under certain soil conditions, particularly in acidic or reducing environments [35]. However, CONAMA Resolution No. 420/2009 does not provide a soil prevention value for Mn, so this result should be interpreted as an agronomic and environmental warning rather than as a formal regulatory exceedance [27]. For the elements with available soil prevention values, the highest concentrations observed in amended soils remained below the limits established by CONAMA Resolution No. 420/2009: Cd^2+^ reached 0.198 mg kg^−1^ compared with 1.3 mg kg^−1^; Pb^2+^ reached 1.254 mg kg^−1^ compared with 72 mg kg^−1^; Cr^3+^ reached 0.950 mg kg^−1^ compared with 75 mg kg^−1^; Ni^2+^ reached 1.040 mg kg^−1^ compared with 30 mg kg^−1^; Cu^2+^ reached 1.160 mg kg^−1^ compared with 60 mg kg^−1^; and Zn^2+^ reached 11.500 mg kg^−1^ compared with 300 mg kg^−1^. Thus, although some elements increased substantially in relative terms, their absolute concentrations remained below Brazilian prevention values for soils. Some reductions in detected concentrations, such as those observed for Cu^2+^ in several treatments, may reflect changes in metal recovery during soil extraction after organic matter addition, potentially shifting part of the metal pool into less extractable forms.

The role of microbial communities should also be considered in future assessments of macrophyte-based amendments. In aquatic systems, macrophytes interact with endophytic and rhizospheric microorganisms that can influence nutrient cycling, plant stress tolerance, metal accumulation, and contaminant transformation [36]. After harvest and soil incorporation, macrophyte material may introduce microbial biomass and organic substrates that affect soil microbial activity during decomposition. Although the present study did not characterize microbial communities, this dimension may help explain part of the variation not captured by rSQS and rPPS. Future studies should therefore combine integrated soil and plant indices with direct measurements of decomposition dynamics, metal speciation, organic compound profiles, microbial community composition and ecotoxicological responses.

Taken together, the results support the potential use of selected aquatic macrophyte materials as organic amendments for degraded pasture soils, while also showing that this use requires species-specific and dose-specific evaluation. The independent construction of rSQS and rPPS, followed by their strong positive association, indicates that these indices can be useful complementary tools for summarizing multidimensional soil and plant responses. At the same time, the non-proportional relationship between rSQS and rPPS observed in some treatments, together with the pronounced increase in Mn^2+^, highlights the need for caution when interpreting integrated scores as direct indicators of agronomic safety or mechanistic causality. Therefore, macrophyte-based amendments should be viewed as promising components of nutrient recycling and circular bioeconomy strategies, as also proposed in previous studies on the agricultural reuse of harvested aquatic biomass [5,12,15]. However, their application should be accompanied by chemical characterization, contaminant monitoring and longer-term field validation.

## 5. Conclusions

This study supports the potential use of selected aquatic macrophyte materials as organic amendments for degraded pasture soil under controlled pot-experiment conditions. Macrophyte incorporation generally improved both rSQS and rPPS, and the positive association between these independently constructed indices indicates that improvements in integrated soil quality were generally accompanied by better *U. decumbens* performance. However, the responses varied according to macrophyte species and application dose, indicating that aquatic plant material should not be considered a uniform amendment.

The results also show that agronomic performance and environmental safety depend on the chemical characteristics of the macrophyte material, the soil response after incorporation and the dose applied. Therefore, rather than supporting a direct recommendation of a single species or dose, this study highlights the need for prior characterization of harvested biomass, monitoring of potentially toxic elements in amended soils and evaluation of species-specific responses. The descriptive non-proportional rSQS–rPPS pattern observed for ELDDE, together with the pronounced increase in Mn^2+^, further indicates that macrophyte reuse should be accompanied by caution and site-specific assessment.

Thus, macrophyte-based amendments may contribute to nutrient recycling and circular bioeconomy strategies, but their practical use requires longer-term field studies to evaluate repeated applications, contaminant dynamics, soil–plant interactions and performance under real pasture conditions.

## Figures and Tables

**Table 1:** Factorial ANOVA for rSQS values.

	Sum of Squares	Degrees of Freedom	F	*p* (>F)
Species	0.41035	3	183.1832	<0.0001
Dose	0.38899	2	260.4711	<0.0001
Species × Dose	0.07110	6	15.8701	<0.0001
Residual	0.03584	48		

**Table 2:** Factorial ANOVA for rPPS values.

	Sum of Squares	Degrees of Freedom	F	*p* (>F)
Species	0.7858	3	21.5332	<0.0001
Dose	0.6293	2	25.8638	<0.0001
Species × Dose	0.0680	6	0.9320	0.4809
Residual	0.5839	48		

**Table 3:** Soil potential acidity, represented by H^+^ + Al^3+^, and concentrations of potentially toxic elements after macrophyte incorporation. Values represent soil concentrations, with percentages in parentheses indicating values relative to the unamended control.

	Dose (t ha^−1^)	H^+^ + Al^3+^	Cu^2+^	Mn^2+^	Zn^2+^	Cr^3+^	Ni^2+^	Cd^2+^	Pb^2+^
CONTROL	0	38.2 (100.0%)	1.1 (100.0%)	1.38 (100.0%)	2.7 (100.0%)	0.286 (100.0%)	0.18 (100.0%)	0.032 (100.0%)	0.514 (100.0%)
ELDDE	5	26.6 (69.6%)	0.94 (85.5%)	12.88 (933.3%)	4.7 (174.1%)	0.496 (173.4%)	0.27 (150.0%)	0.062 (193.8%)	0.464 (90.3%)
	10	26 (68.1%)	1 (90.9%)	21.84 (1582.6%)	7.1 (263.0%)	0.612 (214.0%)	0.402 (223.3%)	0.1 (312.5%)	0.646 (125.7%)
	20	24.4 (63.9%)	1.06 (96.4%)	50.8 (3681.2%)	11.36 (420.7%)	0.772 (269.9%)	0.536 (297.8%)	0.138 (431.2%)	0.82 (159.5%)
MYRAQ	5	27.6 (72.3%)	0.98 (89.1%)	17.58 (1273.9%)	4.74 (175.6%)	0.844 (295.1%)	0.52 (288.9%)	0.142 (443.8%)	0.764 (148.6%)
	10	26.6 (69.6%)	1 (90.9%)	30.5 (2210.1%)	6.54 (242.2%)	0.95 (332.2%)	0.62 (344.4%)	0.178 (556.2%)	1.254 (244.0%)
	20	27 (70.7%)	1.12 (101.8%)	61.12 (4429.0%)	11.5 (425.9%)	0.558 (195.1%)	0.594 (330.0%)	0.106 (331.3%)	1.25 (243.2%)
PIIST	5	28.6 (74.9%)	1.16 (105.5%)	2.22 (160.9%)	3.16 (117.0%)	0.354 (123.8%)	0.568 (315.6%)	0.098 (306.2%)	0.614 (119.5%)
	10	21.8 (57.1%)	1.1 (100.0%)	2.84 (205.8%)	3.68 (136.3%)	0.522 (182.5%)	0.77 (427.8%)	0.132 (412.5%)	0.722 (140.5%)
	20	16.6 (43.5%)	1 (90.9%)	5.06 (366.7%)	4.5 (166.7%)	0.864 (302.1%)	0.92 (511.1%)	0.186 (581.2%)	0.756 (147.1%)
SAVAU	5	33.8 (88.5%)	1 (90.9%)	4.72 (342.0%)	2.48 (91.9%)	0.876 (306.3%)	0.942 (523.3%)	0.178 (556.2%)	0.526 (102.3%)
	10	31.6 (82.7%)	1 (90.9%)	7.72 (559.4%)	2.76 (102.2%)	0.942 (329.4%)	1.04 (577.8%)	0.198 (618.8%)	0.548 (106.6%)
	20	28.8 (75.4%)	0.94 (85.5%)	15.18 (1100.0%)	3.18 (117.8%)	0.658 (230.1%)	0.718 (398.9%)	0.148 (462.5%)	0.638 (124.1%)

**Figure A1:**
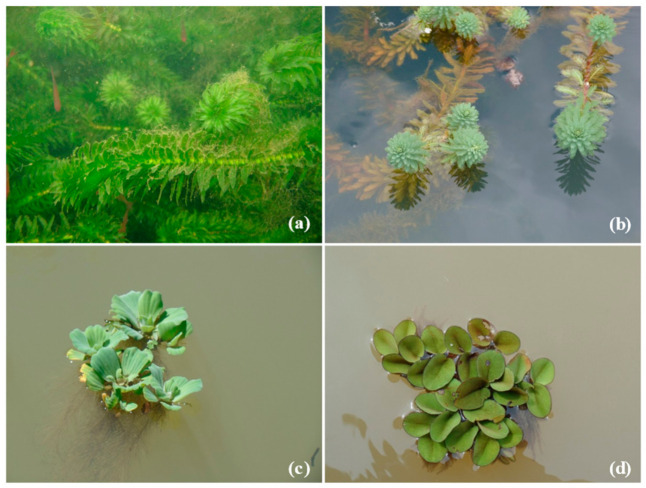
Representative images of the aquatic macrophyte species used in this study: (**a**) ELDDE, (**b**) MYRAQ, (**c**) PIIST and (**d**) SAVAU.

## Data Availability

All analyzed data are presented in the Supplementary Materials.

## Supplementary Materials

The following supporting information can be downloaded at: https://www.mdpi.com/article/10.3390/plants15121788/s1: Supplementary Data S1: Excel file containing experimental soil and plant data used to calculate rSQS and rPPS, a README sheet describing all worksheets, the chemical composition of macrophytes collected from the Santana Reservoir at different sampling times and the final fitted parameters used in the standardization functions for soil and plant indicators.

## Abbreviations

The following abbreviations are used in this manuscript:

rSQS	Relative Soil Quality Score
rPPS	Relative Plant Performance Score
SAVAU	*Salvinia auriculata* Aubl.
MYRAQ	*Myriophyllum aquaticum* (Vell.) Verdc.
ELDDE	*Egeria densa* Planch.
PIIST	*Pistia stratiotes* L.

## Appendix A. Additional Methodological Details

### Appendix A.1. Additional Details on Macrophyte Species, Experimental Units and Seedlings

The four aquatic macrophyte species used in this study were selected because they are abundant or ecologically relevant in the Santana Reservoir and represent contrasting biological forms. Two species were submerged macrophytes, ELDDE and MYRAQ, whereas two were free-floating species, PIIST and SAVAU. This distinction was considered relevant because submerged and floating species may differ in tissue composition, particularly in the contents of structural compounds such as cellulose, hemicellulose, and lignin which may influence decomposition dynamics and agronomic behavior after soil incorporation [13].

ELDDE is a fully submerged aquatic plant of the family Hydrocharitaceae, forming large conglomerates that are transported by water flow and may cause clogging in hydroelectric turbines and pumping systems. MYRAQ is also a submerged plant, although it produces small emergent shoots above the water surface. It belongs to the family Haloragaceae. This species is likewise carried downstream and causes operational problems in hydropower facilities and intake pumps. PIIST is a free-floating plant of the family Araceae, commonly found in lentic systems and known to cause severe impacts in various types of aquatic environments worldwide. SAVAU is a small aquatic fern of the family Salviniaceae, characterized by rapid proliferation. All species analyzed are native to South America and the floating species have been investigated as potential organic fertilizers in several countries [37,38,39]. Representative photographs of the four macrophyte species used in the experiment are shown in Figure A1.

The experimental units consisted of black plastic pots with a diameter of 12 cm, a height of 20 cm and a volumetric capacity of 1.7 L. Each pot contained 1 L of air-dried fine soil, corresponding to 1290 g. The pots had four drainage holes at the bottom, which were covered with porous paper to prevent soil loss while allowing the release of gases produced during macrophyte decomposition. No water loss occurred through the drainage holes because irrigation was controlled gravimetrically, maintaining soil moisture between 50% and 70% of field capacity.

The application rates, expressed as t ha^−1^ of dry matter, were converted to pot-scale amounts using an equivalent soil mass approach. For this conversion, a 0–10 cm soil layer was considered for 1 ha. Since 1 ha corresponds to 10,000 m^2^, this layer represents a soil volume of 1000 m^3^. The apparent density of the soil was 1.29 g cm^−3^, equivalent to 1290 kg m^−3^. Therefore, the estimated soil mass in the 0–10 cm layer was 1,290,000 kg ha^−1^. Each pot contained 1290 g of air-dried fine soil, corresponding to 1.29 kg, which represents 1/1,000,000 of the estimated soil mass per hectare. Accordingly, the field application rates of 5, 10, and 20 t ha^−1^ dry matter corresponded to 5, 10, and 20 g of dry macrophyte material per pot, respectively.

*U. decumbens* seeds were purchased from Sementes Jaboticabal Ltda. and had a germination capacity of 85%. Seedlings were produced in polystyrene trays designed for vegetable seedling production, using the commercial horticultural substrate Tropstrato HT. The seedlings were transplanted into the experimental pots when they had developed three or four fully expanded leaves.

### Appendix A.2. Calculation of rSQS and rPPS

The rSQS and rPPS calculations were based on the indicator scoring approach adapted from [20]. Each indicator was standardized to a 0–1 scale according to its desirability profile: “more is better”, “less is better” or “optimal value”. For indicators classified as “more is better” or “less is better”, the standardized score was calculated using Equation A1 developed by [22] for engineering systems:

(A1) $$v=\frac{1}{1+{\left(\frac{B-L}{x-L}\right)}^{2S(B+x-2L)}}$$

where v is the standardized score, x is the observed value of the indicator, B is the critical or base value at which the standardized score equals 0.5, L is the lower limit of the indicator and S is a slope parameter controlling how rapidly the score changes around B. In this study, L was fixed at zero for all monotonic scoring functions.

The parameters of the monotonic scoring functions were estimated separately for each indicator. First, a parametric normal cumulative distribution function (CDF) was estimated using the observed mean and standard deviation of the indicator. For each observed value, its cumulative probability under this estimated distribution was calculated. These pairs of observed values and cumulative probabilities were then used as a reference to calibrate the scoring function.

The estimated normal CDF was not used directly as the final score. Instead, it served as a reference sigmoid curve for parameter estimation, allowing the observed distribution of each indicator to guide the shape of the scoring function. A logistic model was fitted to approximate this reference relationship and to obtain the parameters required by Equation A1. The fitted logistic midpoint was used as the critical value B. The fitted logistic slope was transformed into the parameter S. For indicators classified as “more is better”, S was assigned a positive sign, whereas for indicators classified as “less is better”, S was assigned a negative sign.

For indicators classified as “optimal value”, the standardized score was calculated using a Gaussian scoring function:

(A2) $$v=\exp\left(\frac{-{\left(x-Cen\right)}^{2}}{2{\left(Wid\right)}^{2}}\right)$$

where v is the standardized score, x is the observed value of the indicator, Cen is the center of the Gaussian function and Wid is the width parameter controlling how rapidly the score decreases as values move away from the optimum. This formulation assigns a maximum score of 1 at the center of the curve.

For optimal-value indicators, the target value ($x_{opt}$) was defined a priori based on agronomic or chemical criteria rather than estimated directly from the observed data. Values below the optimum were initially treated as a “more is better” response, whereas values equal to or above the optimum were treated as a “less is better” response. These preliminary monotonic scores were then used to fit the Gaussian scoring function. The Gaussian model was initialized with the center equal to the predefined optimum, amplitude fixed at 1 and width equal to the observed standard deviation of the indicator. The fitted center and width were recorded as Cen and Wid, respectively.

After standardization, all indicators assumed values between 0 and 1. Equal weights were assigned to all indicators within each score to avoid introducing subjective prioritization among variables. Therefore, for a score composed of *n* indicators, each indicator received a weight of $w_{i}=1/n$. The final score was calculated as the weighted sum of the standardized indicator values:

(A3) $$Score=\sum_{i=1}^{n}v_{i}w_{i}$$

where $v_{i}$ is the standardized score of the i-th indicator, $w_{i}$ is its corresponding weight and n is the number of indicators included in the score. The same formulation was used for both rSQS and rPPS, differing only in the set of indicators included in each score. The desirability profile and fitted parameters used for each indicator are provided in Supplementary Material.

Higher rSQS or rPPS values indicate better relative soil quality or plant performance within the evaluated dataset. Because both scores are relative, they should be interpreted as comparative measures among treatments rather than as absolute measures of soil quality or plant performance.
